# Effects of supplementation with krill oil on blood parameters, hair quality, and fecal microbiota in male beagle dogs

**DOI:** 10.3389/fmicb.2025.1587149

**Published:** 2025-08-01

**Authors:** Wencan Wang, Ling Xu, Yong Cao, Guo Liu, Yan Zhang, Xi Wang, Xin Mao

**Affiliations:** ^1^Chongqing Sweet Pet Products Co., Ltd., Chongqing, China; ^2^Guangdong Provincial Key Laboratory of Nutraceuticals and Functional Foods, College of Food Science, South China Agricultural University, Guangzhou, China; ^3^Guangdong Laboratory for Lingnan Modern Agriculture, Guangzhou, China; ^4^College of Light Industry and Food, Zhongkai University of Agriculture and Engineering, Guangzhou, China; ^5^Department of Animal Nutrition and Feed, College of Biological Engineering, Sichuan Water Conservancy Vocational College, Chengdu, China

**Keywords:** krill oil, antioxidant, immunomodulatory, hair quality, fecal microbiota, dogs

## Abstract

Krill oil (KO) is a bioactive substance with antioxidant and immunomodulatory functions. This study investigated the impact of administering snacks supplemented with 0.5% KO for 8 weeks on the blood parameters, hair quality, and fecal microbiota in dogs. KO was determined to elevate the activities of serum superoxide dismutase (SOD), catalase (CAT), glutathione peroxidase (GSH-Px), and immunoglobulin G (IgG) level. Concurrently, KO markedly diminished serum tumor necrosis factor-α (TNF-α), interleukin-1β (IL-1β), interleukin-8 (IL-8), and malondialdehyde (MDA) levels in dogs. The KO group displayed a considerably higher ratio of ideal scores and thinner hair scales for newborn hair, as well as a significant increase in total amino acid and methionine (Met) content in comparison to the control group. Furthermore, 16S rRNA sequencing revealed the changes in the composition of fecal microbiota after KO feeding. KO significantly affected the α and β diversity of canine fecal microbiota. Moreover, KO augmented the relative abundance of Bacteroidota, Actinobacteriota, and Proteobacteria at the phylum level, and it increased the relative abundance of *Allobaculum*, *Bifidobacterium*, *Prevotella_9*, *Collinsella*, and *Turicibacter* at the genus level. In summary, this study provides valuable insights to further understand the role of KO in promoting canine health.

## 1 Introduction

As the economy rapidly develops, the population of individuals owning pets, particularly dogs and cats, is steadily increasing. Pets significantly contribute to human life by providing emotional support, enhancing mental health, and improving overall quality of life. For many pet owners, pets are not merely companions but also essential family members (Shepherd, 2008). The primary aim of pet nutrition is to enhance physical appearance, promote health, and prolong life expectancy (Buchanan et al., 2011). Currently, the health of pet hair has become a significant concern for pet owners. Superior hair is regarded not only as an external sign of pet health but also a vital evaluation of the nutritional value of pet foods (Li and Wu, 2023). For companion animals valued for their appearance, superior hair can significantly enhance their aesthetics thereby reinforcing the bond between humans and pets. In addition, the intestinal tract is the site of nutrient digestion and absorption and an important immune organ in pets, its health is a growing concern for pet owners (Alessandri et al., 2020). In particular, microbiota is being emphasized for its role in maintaining the intestinal barrier and immunity (Thursby and Juge, 2017). Currently, there is a significant increase in the number of dogs being fed worldwide. The number of pet dogs in Europe exceeds 100 million, 65 million households in the United States own dogs, and 50 million dogs in China are fed by urban households. Therefore, enhancing the hair quality of dogs and maintaining its intestinal health is essential.

Krill oil (KO), derived from Antarctic krill, exists as a naturally functional product that is high in *n*-3 polyunsaturated fatty acids (PUFAs), of which 31.13% of docosahexaenoic acid (DHA) and 14.87% of eicosapentaenoic acid (EPA) are in the form of phospholipids (Colletti et al., 2021; Xie et al., 2019), making them higher bioavailability (Ulven and Holven, 2015). Accumulating evidence demonstrated KO’s multifaceted physiological regulation potential through its antioxidant and anti-inflammatory properties. Studies revealed KO’s capacity to mitigate oxidative damage in diabetic/obese mice while improving cognitive function via oxidative stress reduction (Choi et al., 2024; Li et al., 2018; Meng et al., 2024; Xiong et al., 2018). Moreover, KO exhibited potent anti-inflammatory activity, suppressing tumor necrosis factor-α (TNF-α) secretion in both lipopolysaccharide (LPS)-stimulated human acute monocytic leukemia cells (THP-1) and rat peritoneal macrophages, with consistent efficacy in mice inflammation models (Bonaterra et al., 2017; Vigerust et al., 2013; Zadeh-Ardabili and Rad, 2019). Furthermore, KO administration downregulated interleukin-related gene expression in colitis-induced rats and reduced circulating C-reactive protein levels in chronic inflammation patients (Deutsch, 2007; Grimstad et al., 2012). Moreover, it is suggested that KO ameliorated the inflammatory state of the intestine through anti-inflammatory and antioxidant effects, as well as modulation of the intestinal microbiota in mice (Zhou et al., 2021). Moreover, KO may regulate microbiota balance and enhance intestinal barrier integrity in mice, as indicated by a rise in beneficial bacteria and a decrease in endotoxin permeability (Du et al., 2024). In addition, KO restored intestinal dysbiosis in parasite-infected pigs and colitis mice while exerting anti-inflammatory effects (Liu et al., 2020).

Currently, limited research has been performed on the efficacy of KO in enhancing skin or hair health in both humans and mice. Research indicates that KO can reduce moisture loss and enhance elasticity in human skin (Handeland et al., 2024). Moreover, the combination of KO and astaxanthin effectively attenuated ultraviolet-induced skin photoaging in mice, indicating its potential for the preservation of skin health (Kim et al., 2023; Komatsu et al., 2017). Although many studies on other species, like mice, rats, and pigs, have been demonstrated the physiological advantages of KO (Choi et al., 2024; Du et al., 2024; Grimstad et al., 2012; Huang et al., 2022; Liu et al., 2024), existing studies regarding its effects on canine health have mainly focused on arthritis alleviation (Buddhachat et al., 2017; Soontornvipart et al., 2024). There remains a deficiency of study concerning the effects of KO on improving the hair health, as well as on antioxidant and immunomodulatory functions, and fecal microbiota in dogs. This research focused on exploring the impact of KO on antioxidant and immunomodulatory functions, hair quality, and fecal microbiota in male dogs. The findings will establish a scientific foundation for the continued advancement and utilization of KO as a bioactive ingredient in functional pet foods.

## 2 Materials and methods

### 2.1 Snacks, animals, and feeding

Based on common canine supplements on the market, KO is usually added at approximately 1–1.5%. Considering that snacks are fed in larger amounts than supplements, the experimental snacks used in this experiment contained 0.5% KO. The ingredient and chemical composition of snacks is presented in Table 1. The content of DHA and EPA in KO is 15.26% and 26.05%, respectively, and the other fatty acid composition of KO is presented in Table 2.

**Table 1:** The ingredients and chemical composition of experimental snacks.

Ingredients	Content (per 100 g snacks, as-fed basis)	
	CONT snacks	KO snacks
Water	1.15	1.14
Rice flour	44.74	44.51
Fishmeal	8.95	8.9
Chicken liver powder	2.76	2.75
Chicken digest	3.91	3.89
Chicken meal	2.53	2.52
Chicken fat	2.75	2.74
Glycerol	9.20	9.16
Maltose syrup	14.50	14.42
Xanthan gum	0.55	0.55
Calcium carbonate	8.05	8.01
Potassium sorbate	0.23	0.23
Calcium propionate	0.34	0.34
Sodium hexametaphosphate	0.21	0.21
Taurine	0.10	0.10
Vitamin E	0.02	0.02
KO	-	0.5
Proximate analysis (DM basis, %)		
Crude protein	12.52	12.89
Crude fat	4.55	4.91
Crude fiber	1.25	1.19
Ash	11.94	12.03
Moisture	11.01	10.85
DHA	ND	0.05
EPA	0.01	0.06
Gross energy (Kcal/kg)	3600.52	3628.39

CONT, control group; KO, krill oil; DM, dry matter; DHA, docosahexaenoic acid and EPA, eicosapentaenoic acid. ND indicates that the result is below the lower limit of detection.

**Table 2:** The fatty acid composition of KO.

Fatty acid	Content (%)
C11:0	0.00
C12:0	0.14
C13:0	0.05
C14:0	9.04
C14:1	0.12
C15:0	0.37
C15:1	0.00
C16:0	25.46
C16:1	4.77
C17:0	0.15
C17:1	0.00
C18:0	1.21
C18:1n9t	0.08
C18:1n9c	10.39
C18:2n6t	0.00
C18:2n6c	1.79
C20:0	0.09
C18:3n6	0.11
C20:1	0.65
C18:3n3	1.75
C21:0	0.03
C20:2	0.16
C22:0	0.08
C20:3n6	0.13
C22:1n9	0.76
C20:3n3	0.31
C20:4n6	0.69
C23:0	0.03
C22:2	0.11
C24:0	0.01
C24:1	0.20

Twelve healthy adult male unneutered beagle dogs with an average body weight (BW) of 14.2 ± 0.17 kg and age of 3.0 ± 0.00 years old were selected for this study. One month prior to the experiment, all dogs were immunized with rabies and penta-vaccine, and dewormed internally and externally. The dogs were randomly allocated to the control group (CONT group, *n* = 6) and krill oil group (KO group, *n* = 6) with BW of 14.1 ± 1.56 and 14.2 ± 2.07 kg, respectively. According to similar studies, all dogs were individually kept in cages (1.2 m length × 1.2 m width × 1.4 m height) that were routinely cleaned and disinfected, and the dogs were guaranteed outdoor exercise and socialization with each other 2 times a day for an hour each time (Geary et al., 2022; Yang et al., 2022). The temperature of the kennel was regulated at 23–25°C and humidity at 50–60%, and the dogs were provided with sufficient light and water.

A dry and extruded commercial maintenance complete diet (5.9% ash, 20.88% crude protein, 8.4% crude fat, and 3.5% crude fiber, gross energy 4,149 Kcal/kg) for medium-size dogs was purchased from Jiangsu Xietong Inc., Nanjing, China. All dogs were fed two meals daily (8:00 am and 4:00 pm) with each meal comprising 150 g diets, which exceeds the nutrient requirements of adult dogs recommended by the FEDIAF. Moreover, a dog’s daily intake of snack calories should not exceed 10% of the calories in the diet. Two hours post-maintenance diet, the dogs in both groups received 15 g of control snacks and 15 g of experimental snacks, respectively. The study duration was 56 days (8 weeks), during which BW and feed intake were recorded weekly. All the experiments followed institutional guidelines of South China Agricultural University and approved by the Institutional Animal Ethics Committee of South China Agricultural University (Permit number: 2025E011).

### 2.2 Hair sample collection and analysis

The hair on the dorsal region of the dogs was shaved 1 day prior to the experiment (Day 0). The hair scoring method established by Rees et al. (2001) outlines the criteria: 1 represents dull, coarse, and dry features; 2 indicates medium reflectivity and softness; 3 denotes high reflectivity and very soft; 4 corresponds to medium softness and greasiness; and 5 signifies a very greasy quality. A score of 3 is considered the ideal. The hair scoring was performed by three observers who were blinded to the experimental treatment. The microstructural characteristics of the hair scales were analyzed using scanning electron microscopy (SEM, Hitachi, SU8100, Tokyo, Japan). Meanwhile, 50 mg of hair sample was preserved on dry ice in a collection tube and assessed for free amino acid content using Ultra Performance Liquid Chromatography (UPLC, APExBIO Technology LLC, Shanghai, China). On Day 56, newborn hair samples were collected from the same region of the dog’s back, and their lengths were measured. Similarly, the newborn hair was also scored, analyzed using SEM, and measured for amino acid content.

### 2.3 Blood sample collection and analysis

Blood sample of dogs was obtained in a fasting state on Day 0 and Day 56, following the previously established method (Wang et al., 2024). Briefly, the hair on the forelimb of dogs was shaved to expose the saphenous vein. After disinfection with iodophor, a blood collection needle was inserted into the saphenous vein to draw 3 mL of blood. Of these, 1 mL was transferred to a lithium heparin anticoagulation tube for automated biochemical analysis (Seamaty, SMT-120VP, Chengdu, China) of γ-glutamyltransferase (GGT), alanine aminotransferase (ALT), aspartate aminotransferase (AST), total bilirubin (TB), serum urea (SUR), serum creatinine (SCR), uric acid (UA), and inorganic phosphorus (PI), while the other 2 mL were centrifuged at 1100 × *g* for 10 min to isolate the serum, which was immediately stored at −20°C for subsequent testing.

### 2.4 Serum biochemistry and enzyme-linked immunosorbent assay (ELISA) analysis

The activities of superoxide dismutase (SOD, Beijing Boxbio Science & Technology Co., Ltd., Beijing, China), catalase (CAT, Beijing Boxbio Science & Technology Co., Ltd., Beijing, China), and glutathione peroxidase (GSH-Px, UpingBio Technology Co., Ltd., Hangzhou, China), as well as the content of malondialdehyde (MDA, Nanjing Jiancheng Bioengineering Institute, Nanjing, China), were assessed following the manufacturers’ protocols. The quantification of serum immunoglobulin G (IgG), immunoglobulin M (IgM), immunoglobulin A (IgA), TNF-α, interleukin-8 (IL-8), and interleukin-1β (IL-1β) were performed using specific ELISA kits (Byabscience Biotechnology Co., Ltd., Nanjing, China).

### 2.5 Fecal sample collection and 16S rRNA sequencing

After the dogs were fasted overnight, feces were collected from the catch tray in the morning of Day 56 prior to feeding the diets. The feces were transferred to sterile collection tubes and immediately preserved at −80°C.

Fecal genomic DNA was extracted utilizing the Stool DNA Kit (Tiangen Biotech, Beijing, China) and then mixed with the primer pairs (341F: 5’-CCTAYGGGRBGCASCAG-3′; 806R: 5’-GGACTACNNGGGGTATCTAAT-3′) and Phusion^®^ High-Fidelity PCR Master Mix (New England Biolabs, Ipswich, MA, United States) to generate PCR amplification products. Next, the amplification products were purified and the NEBNext Ultra II DNA Library Prep Kit (New England Biolabs, Ipswich, MA, United States) was used to construct the sequencing libraries. The quality of the libraries was assessed via q-PCR and Qubit (Thermo Fisher Scientific, Waltham, MA, United States), followed by 16S rRNA sequencing using the NovaSeq6000 platform (Novogene Co., Ltd., Beijing, China). The raw data underwent the removal of barcode and primer sequences, followed by splicing of the reads using FLASH software (version 1.2.11, http://ccb.jhu.edu/software/FLASH/) to produce raw tags (Magoc and Salzberg, 2011). Subsequently, Cutadapt software was employed to align the reverse primer sequences and eliminate residual sequences, thereby reducing interference in the subsequent analysis (Martin, 2011). The raw tags were further processed via Fastp software (version 0.23.4) to produce clean tags (Bokulich et al., 2013), and the tags sequences were used to eliminate chimeric sequences by comparison with the species annotation database (Silva database, https://www.arb-silva.de/ for 16S/18S) to yield effective tags. The QIIME2 software (version 2023.05) was used to perform α and β diversity analysis, where α diversity includes Chao1, Shannon, and Simpson indices and β diversity is principle coordinate analysis (PCoA) analysis.

### 2.6 Statistical analysis

All data underwent testing for normal distribution and variance homogeneity before statistical analysis. The Student’s *t*-test (SPSS, version 28.0) was selected for data analysis, with the exception of feed intake and hair score, which used a non-parametric test. The results were presented as mean ± standard error, with significance determined at a *p*-value < 0.05.

## 3 Results

### 3.1 The physiological performance of dogs

Throughout the experiment, all serum biochemical indicators associated with liver and kidney function were within the standard range, suggesting that the dogs were maintained in a healthy condition (Supplementary Table 1). All dogs received snacks during the experiment and no significant variations in BW and feed intake of maintenance complete diet and snacks were observed between the two groups (*p* > 0.05, Supplementary Figure 1).

### 3.2 Serum antioxidant and immune indices

**Figure 1:**
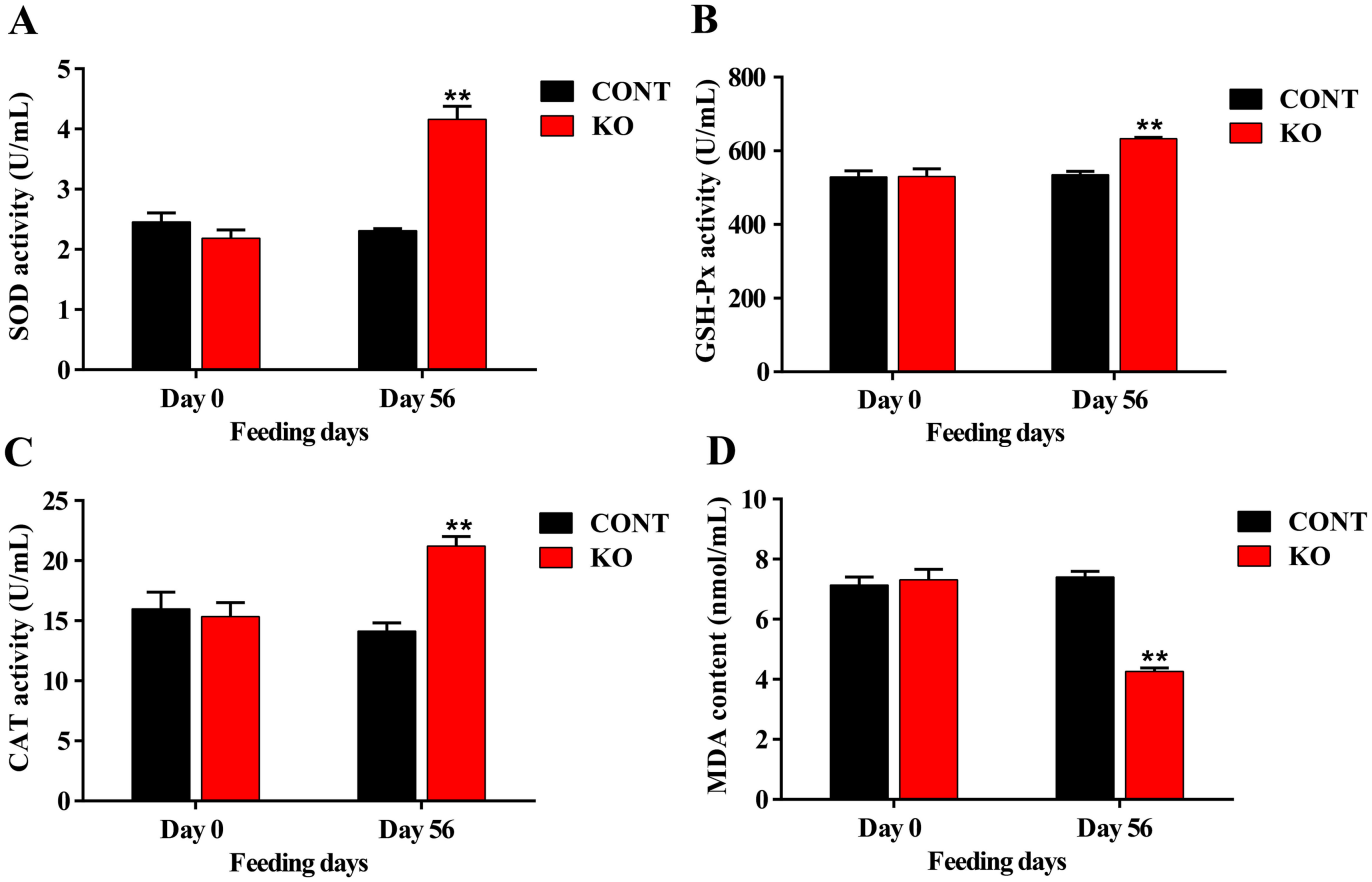
The activities of serum SOD **(A)**, GSH-Px **(B)**, CAT **(C)**, and the content of MDA **(D)**. ^**^p < 0.01.

**Figure 2:**
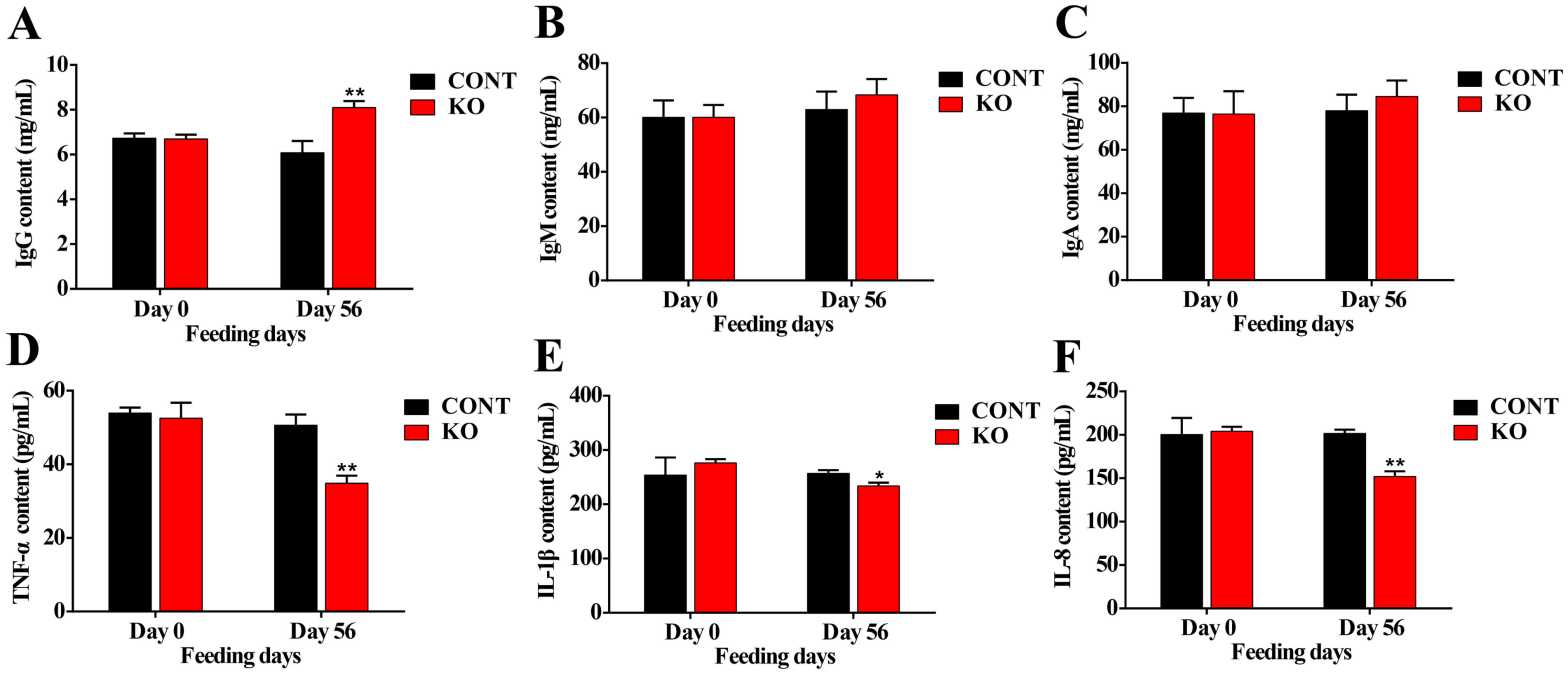
The levels of serum IgG **(A)**, IgM **(B)**, IgA **(C)**, TNF-α **(D)**, IL-1β **(E)**, and IL-8 **(F)**. ^*^p < 0.05; ^**^p < 0.01.

The KO group demonstrated significantly higher activities of serum SOD, GSH-Px, and CAT (*p* < 0.01, Figures 1A–C), and a greatly lower MDA level (*p* < 0.01, Figure 1D) than the CONT group after 8 weeks. Moreover, the IgG content was significantly elevated (*p* < 0.01, Figure 2A). Despite the absence of a significant difference in IgA and IgM levels between the both groups, a rising was observed in the KO group (*p* > 0.05, Figures 2B,C). Furthermore, the KO group exhibited significantly lower levels of TNF-α, IL-1β, and IL-8 in comparison to the CONT group (*p* < 0.05, Figures 2D–F).

### 3.3 Hair quality and microscopic characterization

The two groups showed no significant variation in hair score and length on Day 0 and Day 56 (*p* > 0.05, Table 3). The initial ideal hair ratio was greater in the CONT group than in the KO group (55.56% vs. 50%). After the experiment, the ideal ratio decreased to 50% in the CONT group and escalated to 72.22% in the KO group (Table 3).

**Table 3:** The hair quality parameters in dogs.

Items	CONT	KO	*p-*value
Hair length (mm)			
Day 0	0.00	0.00	–
Day 56	19.5 ± 0.45	20.5 ± 0.21	0.067
Hair score			
Day 0	2.8 ± 0.19	2.7 ± 0.20	0.589
Day 56	2.8 ± 0.19	3.1 ± 0.13	0.310
Ideal ratio			
Day 0	55.56%	50.00%	–
Day 56	50.00%	72.22%	–

**Figure 3:**
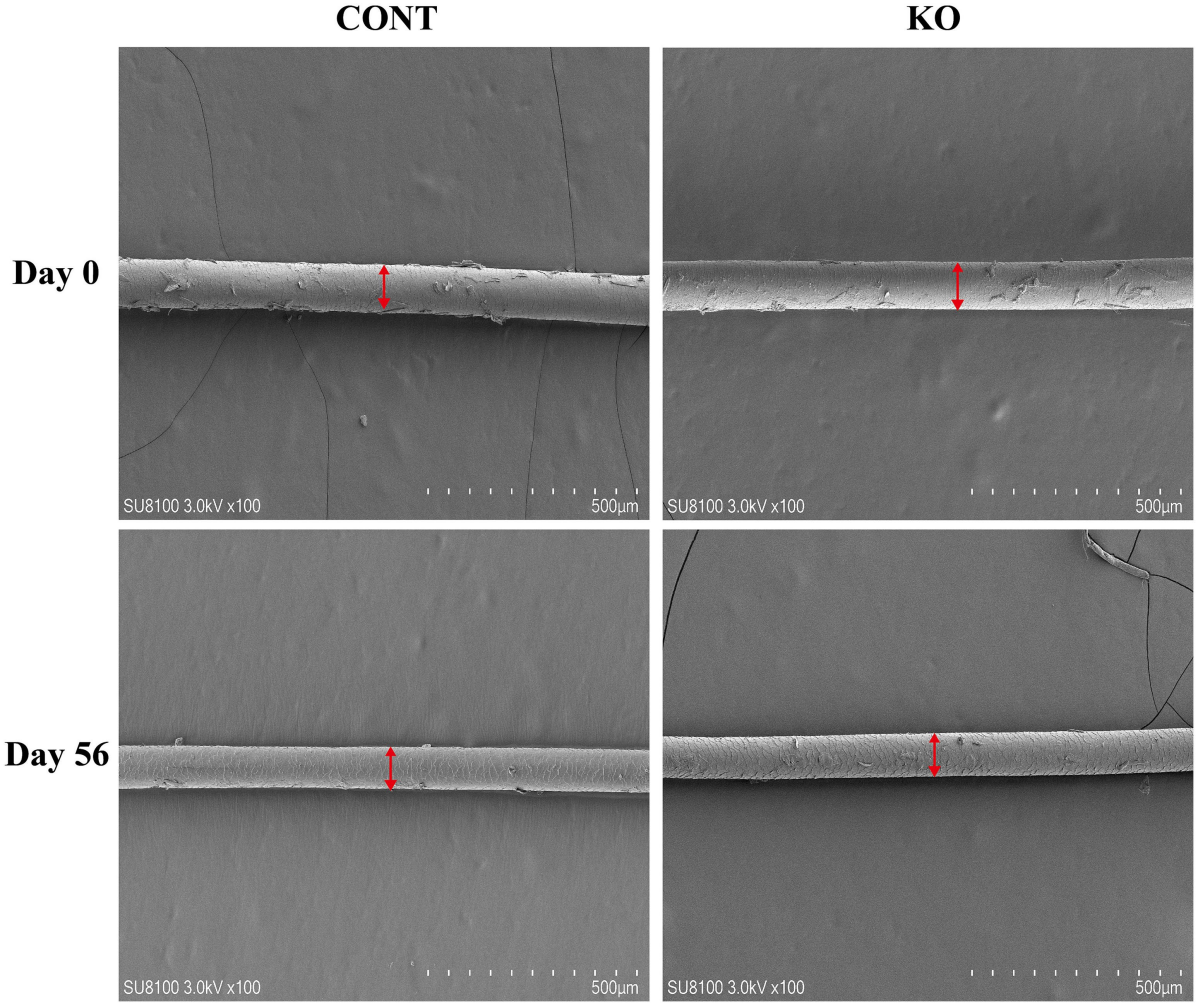
The microscopic demonstration of hair diameter under SEM.

**Figure 4:**
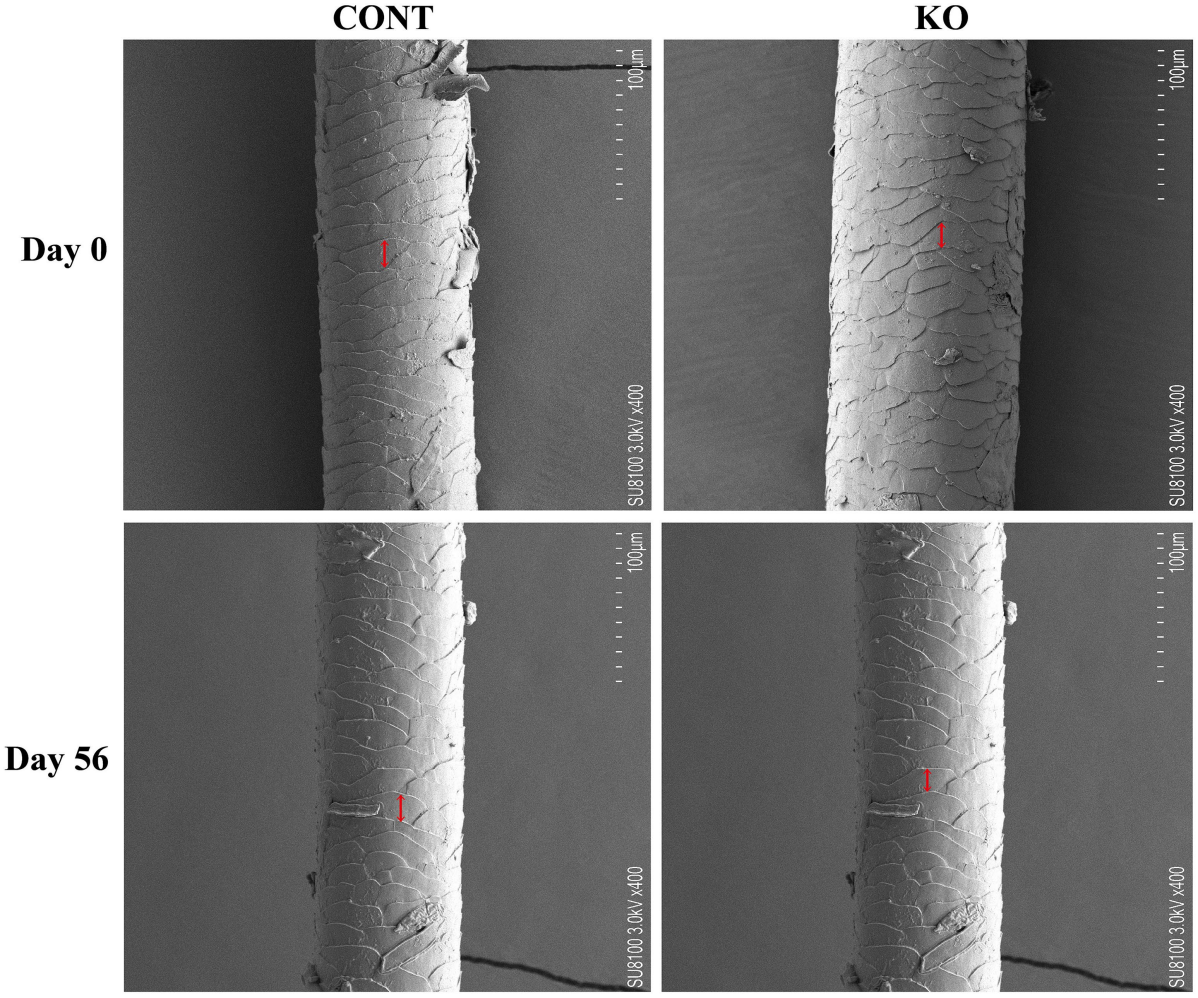
The microscopic demonstration of hair scale height under SEM.

**Figure 5:**
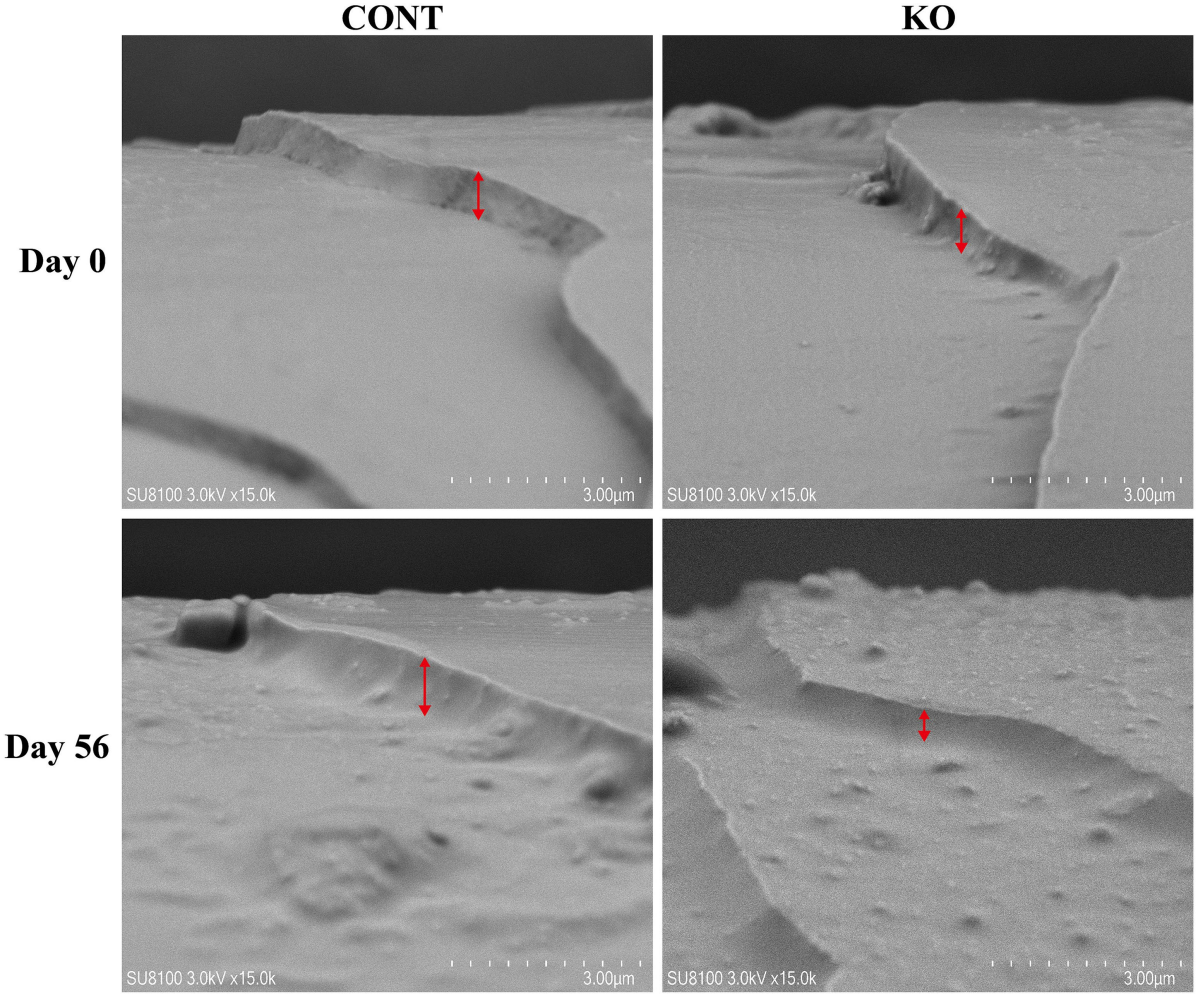
The microscopic demonstration of hair scale thickness under SEM.

Figures 3–5 show the electron microscopic performance of hair diameter, scale height, and scale thickness, respectively. Electron microscopy analyses revealed no significant difference in the hair diameter and scale height (*p* > 0.05, Table 4) between the two groups on Day 0. Nevertheless, the scale thickness was notably reduced in the KO group compared to the CONT group on Day 56 (*p* < 0.01, Table 4).

**Table 4:** Microstructural parameters of hair in dogs.

Items	CONT	KO	*p*-value
Hair diameter (μm)			
Day 0	95.3 ± 5.39	108.1 ± 12.39	0.374
Day 56	113.4 ± 12.41	101.2 ± 10.04	0.467
Scale height (μm)			
Day 0	12.7 ± 1.11	11.7 ± 1.01	0.545
Day 56	12.7 ± 0.70	12.2 ± 0.92	0.704
Scale thickness (μm)			
Day 0	0.7 ± 0.02	0.7 ± 0.03	0.657
Day 56	0.7 ± 0.03	0.5 ± 0.01	<0.01

### 3.4 Hair amino acid content

No significant difference was observed in total amino acid and Met content on Day 0 between the two groups. However, the content of total amino acid and Met in the KO group was greatly higher than the CONT group on Day 56 (*p* < 0.05, Table 5).

**Table 5:** The content of total amino acid and Met in the hair of dogs.

Items	CONT	KO	*p*-value
Total amino acid (μg/g)			
Day 0	1076.0 ± 160.62	1135.5 ± 26.86	0.724
Day 56	1195.3 ± 74.20	1560.6 ± 135.40	0.046
Met (μg/g)			
Day 0	3.2 ± 1.52	2.8 ± 0.40	0.662
Day 56	3.2 ± 0.23	6.6 ± 0.77	<0.01

### 3.5 Changes in fecal microbiota

A total of 1,279,321 raw reads were acquired from all the samples, with 1,078,622 effective reads remaining post-quality control for subsequent analysis. Additionally, the Q30 of all samples exceeded 94%, indicating that the sequencing data were of superior quality and dependable (Supplementary Table 2). A total of 813 operational taxonomic units (OTUs) were identified, with 168 common to both groups, while the CONT and KO groups exhibited 274 and 371 specific OTUs, respectively (Supplementary Figure 2A). In addition, the species dilution curve and hierarchical clustering curve also demonstrated that the data amount was sufficient and the microbiota species richness varied between the two groups (Supplementary Figures 2B,C).

**Figure 6:**
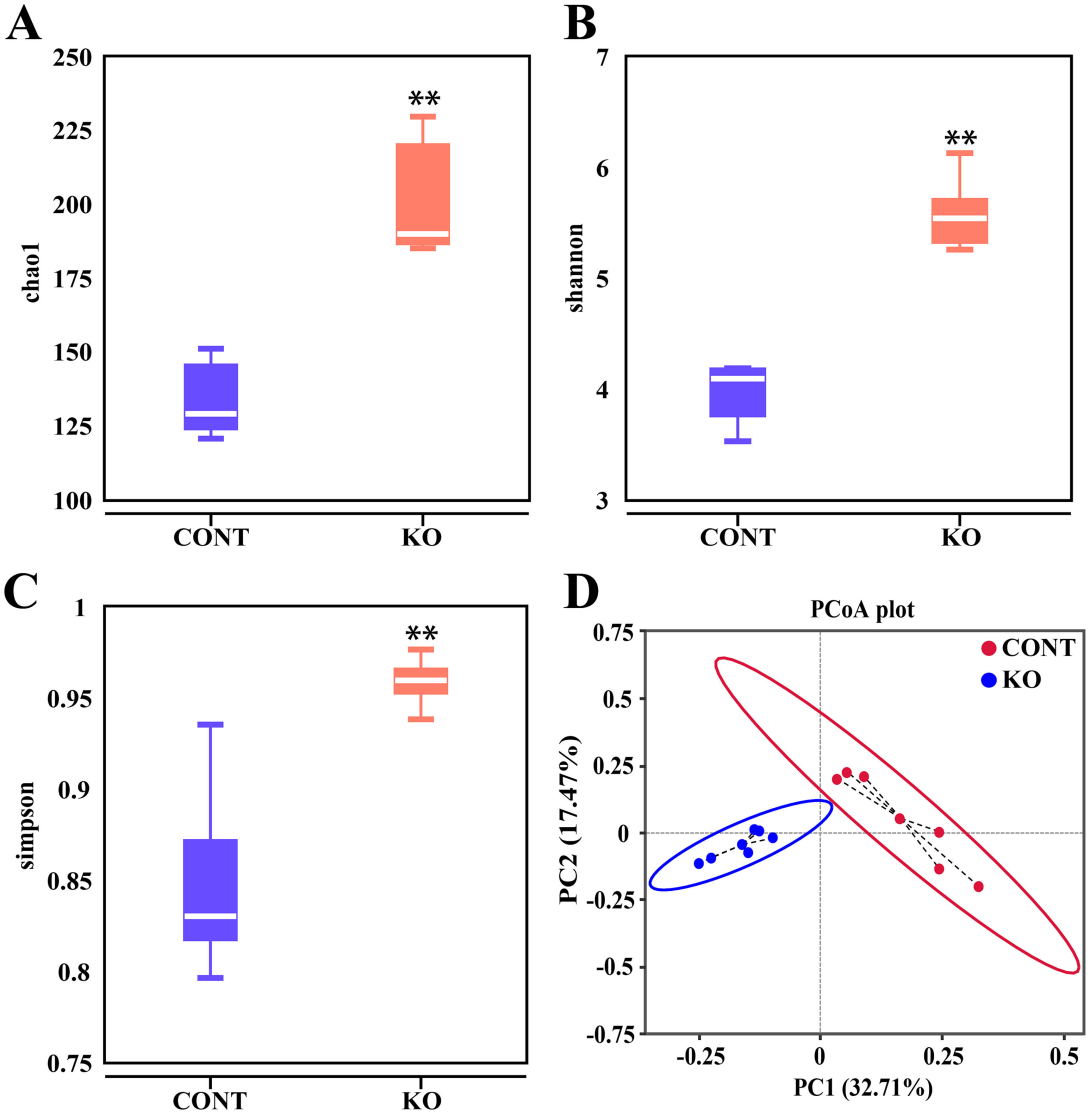
The Chao1 **(A)**, Shannon **(B)**, and Simpson **(C)** indices in α diversity analysis of intestinal microbiota. **(D)** PCoA score plot of fecal microbiota in dogs.

**Figure 7:**
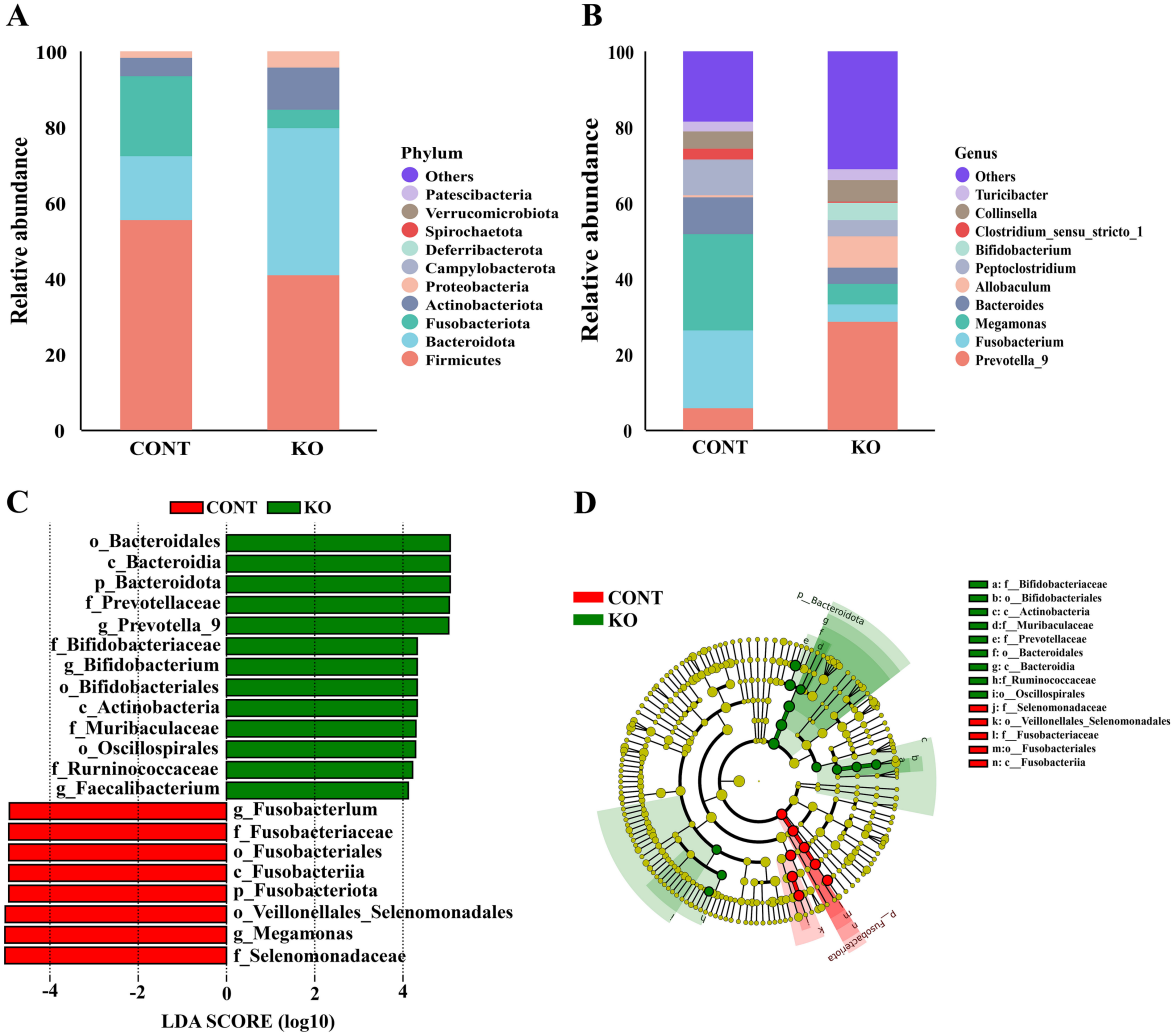
The relative abundance and LEfSe analysis of fecal microbiota. **(A)** The relative abundance of fecal microbiota at the phylum level between the two groups. **(B)** The relative abundance of fecal microbiota at the genus level between the two groups. **(C)** Biomarkers with LDA scores > 4 are shown in the LDA value distribution histograms. **(D)** Cladogram showing a comparison of the microbial profile between the CONT and KO groups.

Microbial diversity analysis indicated that the KO group had significantly higher Chao1, Shannon, and Simpson indices than the CONT group (*p* < 0.01, Figures 6A–C). The PCoA plot revealed a distinct separation of microbiota between the two groups (Figure 6D). Among canine fecal microorganisms, the five main phyla are Firmicutes, Bacteroidota, Fusobacteriota, Actinobacteriota, and Proteobacteria. The KO group had a lower relative abundance of Firmicutes and Fusobacteriota and a higher abundance of Bacteroidota, Actinobacteriota, and Proteobacteria compared to the CONT group (Figure 7A). At the genus level, KO increased the abundance of *Allobaculum, Prevotella_9*, *Bifidobacterium*, *Collinsella,* and *Turicibacter*, while decreasing the abundance of *Fusobacterium*, *Megamonas*, *Bacteroides*, *Peptoclostridium,* and *Clostridium_sensu_stricto_1* (Figure 7B).

Linear discriminant analysis effect size (LEfSe) analysis helped in identifying microbe that might act as biomarkers. The result shows that the *Selenomonadaceae*, *Megamonas*, Veillonellales_Selenomonadales, and Fusobacteriota, etc., were markedly enriched in the CONT group, while the Bacteroidota, *Prevotellaceae*, *Prevotella_9*, and *Bifidobacterium,* etc., were notably enriched in the KO group (Figures 7C,D).

## 4 Discussion

The KO may be an alternative functional ingredient in pet foods because of its physiological regulatory features. We examined the influence of KO on the antioxidant and immune systems of dogs, along with their hair condition and fecal bacteria. Oxidative stress is an important contributor to aging, organ damage, and numerous diseases in animals (Pizzino et al., 2017). The *n*-3 PUFAs in KO primarily consist of DHA and EPA, which function as natural antioxidants (Duo et al., 2024). Studies shown that KO significantly enhanced SOD activity and reduced MDA level in the serum of rats with gastric mucosal injury (Huang et al., 2022). Furthermore, KO was demonstrated to suppress the increase of MDA levels and the reduction of CAT and GSH-Px activities in the liver of high fat-induced obese mice, underscoring its antioxidant properties (Choi et al., 2024). Likewise, KO supplementation markedly enhanced SOD and GSH-Px activities in the serum of d-galactose-induced aging mice (Cheong et al., 2017). Richards et al. (2023) supplemented dogs with camelina oil containing 35.4% *n*-3 PUFAs and found no significant difference in its effect on serum antioxidant indices, but the amount of diet fed and the content of DHA or EPA were unknown. Our findings indicate that snacks containing 0.05% DHA and 0.06% EPA notably increased the activities of serum SOD, CAT, and GSH-Px by 90.83, 38.27 and 19.27%, respectively, and the MDA content decreased significantly by about 70%, which may be related to factors such as DHA and EPA’s content, purity, and absorption efficiency.

As a result of *n*-3 PUFAs’ function in curbing inflammatory factor synthesis and T-cell responses, KO has anti-inflammatory properties in several species (Calder, 2013). Lee et al. (2023) demonstrated that KO decreased serum content of TNF-α, IL-6, and IL-1β in inflamed mice. Researches indicate that KO can markedly alleviate pain and enhance stiffness in individuals with arthritis (Laslett et al., 2020; Stonehouse et al., 2022). Similarly, KO supplementation may enhance pain relief and reduce lameness in canines suffering from arthritis (Soontornvipart et al., 2024). Additionally, a diet high in *n*-3 PUFAs substantially lowered the synthesis of IL-1 and IL-6 after LPS induction, indicating a beneficial anti-inflammatory impact of *n*-3 PUFAs in dogs (LeBlanc et al., 2008). Combarros et al. (2020) discovered that DHA and EPA supplementation reduced blood concentrations of pro-inflammatory agents and alleviated pruritus in dogs. Also, the levels of inflammation-related genes, including heat shock protein 90 (*HSP90*) and *IL-1β*, in canine blood decrease following supplementation with *n*-3 PUFAs-enriched flaxseed oil (Purushothaman et al., 2014). We found that KO greatly diminished the levels of TNF-α, IL-1β, and IL-8 by 50.86, 18.45 and 33.55%, respectively. Moreover, immunoglobulins are crucial elements of humoral immunity in animals, with IgG constituting 75% of blood immunoglobulins, which perform antibacterial, antiviral, and immunomodulatory functions (Navolotskaya, 2014). Our study revealed an increase of about 23% in serum IgG content, reflecting a beneficial impact of KO on the immune system in male dogs, potentially alleviating the body’s inflammatory state to preserve optimal skin function and improve hair quality.

The microstructure of hair, including scale height and thickness, affects its physicochemical properties and sensory characteristics, serving as a crucial metric for assessing hair quality (Mishra et al., 2016). A reduction in the scale thickness and an increase in scale height result in smoother hair (Liu et al., 2017). Some studies have been utilized the scale microstructure to evaluate the hair quality of pets (Guo et al., 2022; Wang et al., 2024; Zhang et al., 2024). Rees et al. (2001) provided dogs with diets containing 0.11% EPA and 0.08% DHA for 1 month, which resulted in a significant improvement in the quality of the dog’s hair. Combarros et al. (2020) found that capsules containing the triglyceride-bound form of 16.5% EPA and 10.3% DHA significantly increased the lipids on the hair shaft of dogs. Although our snacks provided 15 mg DHA and 18 mg EPA, both at lower levels than in the above studies, they still increased hair scores and decreased hair scale thickness thereby achieving improved hair quality in dogs, which may be due to the longer duration of KO supplementation as well as the more efficient absorption of phospholipid-bound DHA and EPA in KO. Notably, KO increased the total amino acid and Met content in the hair of male dogs. Amino acid constitutes the nutritional foundation for sustaining the hair quality in pets, particularly the sulfur-containing amino acid, such as Met. Met has shown the functions to promote hair follicle development and inhibit hair loss in animals (Kubota et al., 2024; Li et al., 2022; Zhao et al., 2023). It is well known that intermediate metabolites are recognized for their role in regenerating Met through the re-methylation pathway to sustain Met homeostasis, while choline, a significant class of methyl donors, supplies methyl for Met cycling. KO contains adequate phosphatidylcholine alongside *n*-3 PUFAs (Xie et al., 2019), prompting us to hypothesize that the high bioavailability of KO may indirectly facilitate the re-synthesis of Met, thereby improving hair quality. Nonetheless, the specific biological mechanisms involved require further investigation.

The intestinal microbiota modulates the host’s physiological functions by regulating metabolism and providing defense against pathogens (Pilla and Suchodolski, 2019). KO modified the structure of intestinal microbiota in dogs. The Firmicutes, Bacteroidota, Fusobacteriota, Actinobacteriota, and Proteobacteria were the most prevalent phyla, aligning with other studies and our previous report (Honneffer et al., 2017; Suchodolski, 2022; Wang et al., 2024). LEfSe analysis indicated that KO supplementation significantly enhanced the abundance of *Bifidobacterium, Muribaculaceae, Ruminococcaceae, Faecalibacterium, Prevotella_9*, *Prevotellaceae*. These microorganisms constitute essential beneficial bacteria in the intestine due to their capacity to produce short-chain fatty acids (Kovatcheva-Datchary et al., 2015; Tan et al., 2016; Zhang et al., 2021; Zhu et al., 2024). *Bifidobacterium* is one of the most common beneficial bacteria in the intestinal tract and has the function of producing antimicrobial agents, synthesizing vitamin B, and enhancing intestinal immunity (Martinez et al., 2013). Strompfova et al. (2014) found that *Bifidobacterium* subspecies reduced fecal *Escherichia coli* levels and increased leukocyte phagocytosis in healthy Beagles. *Muribaculaceae* were recognized as beneficial commensals and their abundance was significantly increased in treated colitis models across multiple species and associated with improved intestinal health in dogs (Cheng et al., 2023; Gao et al., 2021; Liu et al., 2021; Zhao et al., 2025). Importantly, it was discovered that *Muribaculaceae* may contribute to hair follicle development, which is essential for promoting hair health (Yang et al., 2023). *Ruminococcaceae,* pivotal for bile acid metabolism and microbial equilibrium, are markedly reduced in dogs with acute diarrhea and enteritis but restore to baseline post-treatment (Gu et al., 2022; Guard et al., 2015; Zhang et al., 2023). Zhao et al. (2024) and Kang et al. (2024) demonstrated a significant rise in *Ruminococcaceae* abundance following probiotic supplementation in dogs, indicating the crucial role of *Ruminococcaceae* in improving canine intestinal health. *Faecalibacterium* abundance was found to decrease in the intestine of dogs with enteritis, but increased significantly after treatment, suggesting its involvement in the recovery of enteritis and promotes intestinal health (Rossi et al., 2014). Our findings indicate that KO substantially enhances the abundance of these advantageous bacteria, implying a positive impact on intestinal health of male dogs, which may serve as a crucial protective strategy for the organism’s overall health and improve skin function and hair quality. However, this study lacks research on whether there is a direct link between changes in intestinal microbiota and improved hair quality after KO feeding, which needs to be explored further. Furthermore, future validation is needed regarding whether KO has a similar effect on female dogs.

## 5 Conclusion

In conclusion, KO did not significantly influence hair growth, but it notably increased total amino acid and Met content in hair, reduced the thickness of hair scales, and enhanced softness, indicating that KO may be beneficial for canine hair quality. In addition, KO supplementation enhanced the antioxidant capacity and immune function, regulated the composition and structure of canine fecal microbiota, and promoted the proliferation of beneficial bacteria, which is beneficial to the health of the dog’s organism. These findings provide a scientific basis for the use of KO as a functional ingredient in foods to positively promote the health of dogs.

## Data Availability

The original contributions presented in the study are publicly available. This data can be found: https://www.ncbi.nlm.nih.gov/, PRJNA1210795.
