# Supplementary material for: Effects of supplementation with krill oil on blood parameters, hair quality, and fecal microbiota in male beagle dogs
Source: Front Microbiol. 2025 Aug 1;16:1587149. doi: 10.3389/fmicb.2025.1587149 (PMC12354513; doi:10.3389/fmicb.2025.1587149)
Supplement: Supplementary file 1 [file Supplementary_file_1.docx]

Supplementary Material

**Supplementary Table 1**. The dogs’ serum biochemical parameters in CONT and KO groups.

| **Feeding days** | **Groups** | **No.** |  | | **Items** | | | | | |
| --- | --- | --- | --- | --- | --- | --- | --- | --- | --- | --- |
|  |  |  | **GGT**  **(0-10 U/L)** | **ALT**  **(5-125 U/L)** | **AST**  **(0-50 U/L)** | **TB**  **(0-15 μmol/L)** | **SUR**  **(2.5-9.6 mmol/L)** | **SCR**  **(28-159 μmol/L)** | **UA**  **(0-60 μmol/L)** | **PI**  **(0.81-2.19 mmol/L)** |
| Day 0 | CONT | 1 | < 2 | 46 | 19 | < 0.1 | 3.81 | 78.6 | < 10.00 | 1.46 |
|  |  | 2 | < 2 | 70 | 21 | < 0.1 | 3.53 | 72.9 | 11.42 | 1.48 |
|  |  | 3 | < 2 | 61 | 29 | 2.2 | 3.06 | 58.7 | 32.25 | 1.29 |
|  |  | 4 | < 2 | 57 | 16 | 1.0 | 5.25 | 68.8 | < 10.00 | 1.49 |
|  |  | 5 | < 2 | 64 | 34 | 1.4 | 3.65 | 73 | < 10.00 | 0.99 |
|  |  | 6 | < 2 | 53 | 35 | 3.2 | 4.33 | 71.8 | < 10.00 | 0.98 |
|  | KO | 1 | < 2 | 67 | 22 | < 0.1 | 3.60 | 40.6 | < 10.00 | 1.09 |
|  |  | 2 | < 2 | 43 | 29 | < 0.1 | 4.61 | 69.9 | < 10.00 | 1.11 |
|  |  | 3 | < 2 | 78 | 41 | 1.2 | 4.11 | 65.9 | < 10.00 | 1.44 |
|  |  | 4 | < 2 | 72 | 42 | 0.7 | 3.44 | 58.4 | < 10.00 | 1.27 |
|  |  | 5 | < 2 | 58 | 28 | < 0.1 | 3.85 | 48.6 | < 10.00 | 1.53 |
|  |  | 6 | < 2 | 58 | 34 | 0.4 | 3.22 | 48.0 | < 10.00 | 1.49 |
| Day 56 | CONT | 1 | < 2 | 47 | 20 | < 0.1 | 5.66 | 75.5 | < 10.00 | 1.38 |
|  |  | 2 | < 2 | 84 | 24 | < 0.1 | 4.98 | 57.3 | < 10.00 | 1.43 |
|  |  | 3 | < 2 | 69 | 28 | < 0.1 | 4.05 | 59.2 | < 10.00 | 1.21 |
|  |  | 4 | < 2 | 54 | 18 | < 0.1 | 5.40 | 63.4 | < 10.00 | 1.47 |
|  |  | 5 | < 2 | 67 | 45 | < 0.1 | 5.60 | 63.1 | < 10.00 | 0.98 |
|  |  | 6 | < 2 | 53 | 36 | 0.7 | 5.56 | 63.6 | < 10.00 | 1.22 |
|  | KO | 1 | < 2 | 56 | 16 | < 0.1 | 3.74 | 50.2 | < 10.00 | 0.83 |
|  |  | 2 | < 2 | 38 | 17 | < 0.1 | 7.43 | 68.2 | < 10.00 | 1.53 |
|  |  | 3 | < 2 | 57 | 23 | < 0.1 | 4.39 | 60.1 | < 10.00 | 1.43 |
|  |  | 4 | < 2 | 47 | 12 | 0.5 | 2.39 | 48.4 | < 10.00 | 1.20 |
|  |  | 5 | < 2 | 50 | 9 | 1.6 | 2.60 | 48.6 | < 10.00 | 1.28 |
|  |  | 6 | < 2 | 52 | 12 | < 0.1 | 3.83 | 61.4 | < 10.00 | 1.33 |

Note: GGT, ALT, AST, TB, SUR, SCR, UA and PI represent γ-glutamyltransferase, alanine aminotransferase, aspartate aminotransferase, total bilirubin, serum urea, serum creatinine, uric acid and inorganic phosphorus, respectively

**Supplementary Table 2.** The statistical results of 16S rRNA sequencing data.

| **Group** | **Sample** | **Raw reads** | **Effective tags** | **GC (%)** | **Q30 (%)** | **Effective (%)** |
| --- | --- | --- | --- | --- | --- | --- |
| CONT | CONT1 | 105006 | 93454 | 50.66 | 95.40 | 89.00 |
|  | CONT2 | 105989 | 93088 | 54.05 | 94.83 | 87.83 |
|  | CONT3 | 118031 | 101573 | 52.77 | 95.01 | 86.06 |
|  | CONT4 | 104062 | 85728 | 52.57 | 95.18 | 82.38 |
|  | CONT5 | 106710 | 89493 | 50.78 | 95.04 | 83.87 |
|  | CONT6 | 102858 | 82996 | 53.02 | 95.27 | 80.69 |
| KO | KO1 | 103477 | 87816 | 53.70 | 95.62 | 84.87 |
|  | KO2 | 97160 | 81037 | 53.35 | 94.98 | 83.41 |
|  | KO3 | 112004 | 96291 | 52.66 | 95.44 | 85.97 |
|  | KO4 | 102020 | 81267 | 53.10 | 94.70 | 79.66 |
|  | KO5 | 102927 | 78458 | 53.62 | 95.26 | 76.23 |
|  | KO6 | 119077 | 107421 | 51.36 | 95.16 | 90.21 |


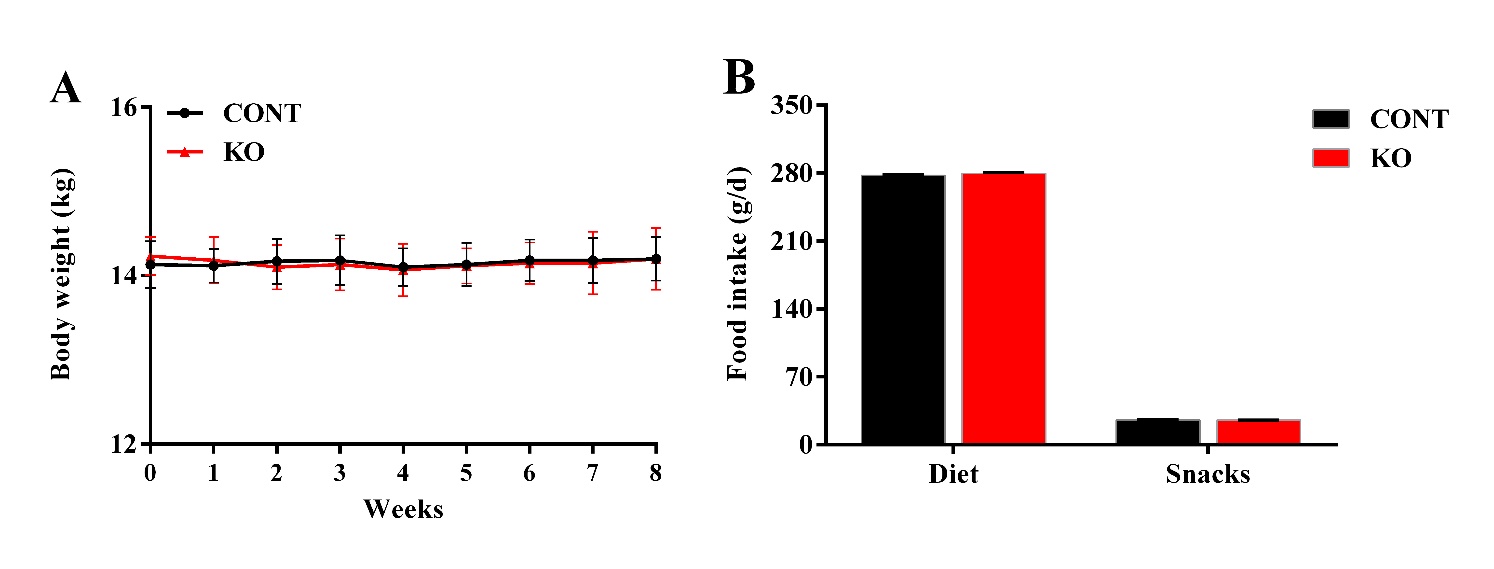


**Supplementary Figure 1**. The dogs’ BW (A) and feed intake (B) in CONT and KO groups.

**
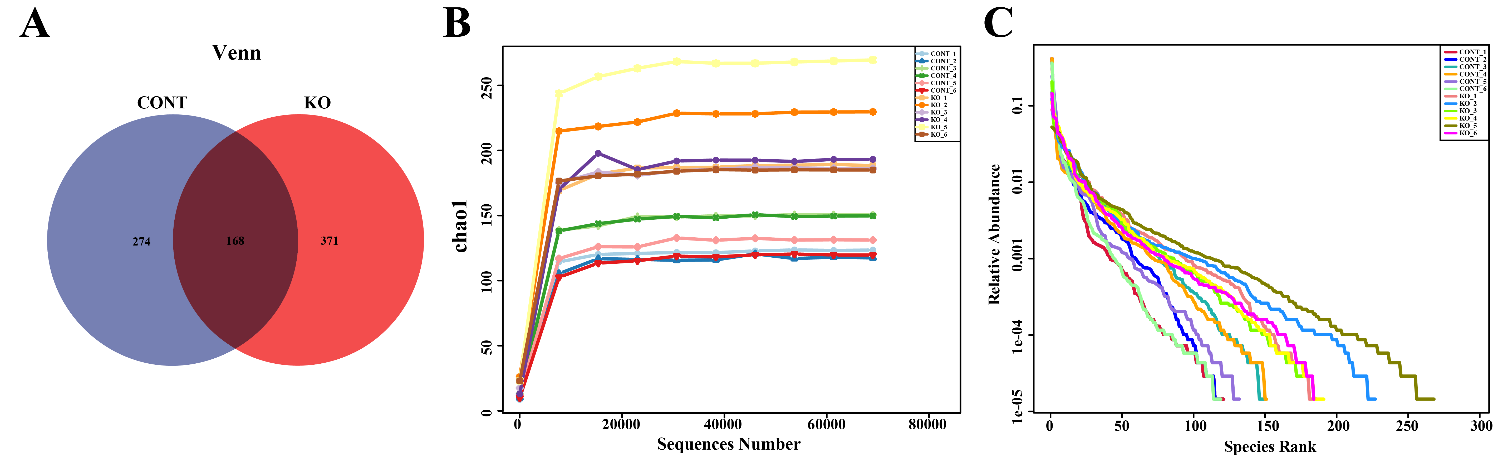
**

**Supplementary Figure 2**. Summary of 16S rRNA sequencing data. (A) Venn diagram of OTUs. (B) The rarefaction curves of samples. (C) The rank abundance curve of samples.
